# Trust of inpatient physicians among parents of children with medical complexity: a qualitative study

**DOI:** 10.3389/fped.2024.1443869

**Published:** 2024-09-27

**Authors:** Tammie Dewan, Andrea Whiteley, Lyndsay Jerusha MacKay, Rachel Martens, Melanie Noel, Chantelle Barnard, Isabel Jordan, Anne Janvier, Sally Thorne

**Affiliations:** ^1^ Department of Pediatrics, University of Calgary, Calgary, AB, Canada; ^2^ Section of Pediatric Hospital Medicine, Alberta Children’s Hospital, Calgary, AB, Canada; ^3^ School of Nursing, Trinity Western University, Langley, BC, Canada; ^4^ Canchild Centre for Childhood Disability Research, McMaster University, Hamilton, ON, Canada; ^5^ Azrrieli Accelerator, University of Calgary, Calgary, AB, Canada; ^6^ Department of Psychology, University of Calgary, Calgary, AB, Canada; ^7^ Independent Researcher, Squamish, BC, Canada; ^8^ Division of Neonatology, Research Center, Unité d’éthique Clinique, Bureau du Partenariat Patients-Familles-Soignants, CHU Sainte-Justine, Montréal, QC, Canada; ^9^ Department of Pediatrics, Bureau de l’éthique clinique (BEC), Université de Montréal, Montréal, QC, Canada; ^10^ School of Nursing, University of British Columbia, Vancouver, BC, Canada

**Keywords:** trust, medical complexity, parents, relationship, patient- and family-centered care, physician, hospital, pediatrics

## Abstract

**Background:**

Trust is a foundation of the therapeutic relationship and is associated with important patient outcomes. Building trust between parents of children with medical complexity (CMC) and physicians during inpatient care is complicated by lack of relational continuity, cumulative (sometimes negative) parent experiences and the need to adjust roles and expectations to accommodate parental expertise. This study's objective was to describe how parents of CMC conceptualize trust with physicians within the pediatric inpatient setting and to provide recommendations for building trust in these relationships.

**Methods:**

Interviews with 16 parents of CMC were completed and analyzed using interpretive description methodology.

**Results:**

The research team identified one overarching meta theme regarding factors that influence trust development: situational awareness is needed to inform personalized care of children and families. There were also six major themes: (1) ensuring that the focus is on the child and family, (2) respecting both parent and physician expertise, (3) collaborating effectively, (4) maintaining a flow of communication, (5) acknowledging the impact of personal attributes, and (6) recognizing issues related to the healthcare system.

**Discussion:**

Many elements that facilitated trust development were also components of patient- and family-centered care. Parents in this study approached trust with inpatient physicians as something that needs to be earned and reciprocated. To gain the trust of parents of CMC, inpatient physicians should personalize medical care to address the needs of each child and should explore the perceptions, expertise, and previous experiences of their parents.

## 1 Introduction

Research and professional consensus support the view that trust is foundational to effective therapeutic encounters (1) and is associated with important outcomes including patient satisfaction, adherence to treatment, clinical outcomes, and patient self-reported health (2, 3). This view is consistent in the adult (1, 2, 4–8) and pediatric context (3, 6, 9, 10), as well as in the inpatient and outpatient settings. Establishment of a trusting relationship is a core component of patient- and family-centered care and provides meaning and importance to the patient-physician relationship (1). Trust between adult patients and physicians has been defined as a physician acting as an advocate for the best interest of their patient, showing genuine concern, and treating their patients with respect and dignity (4). Trust is rooted in vulnerability and safety: the more vulnerable the patient, the higher the likelihood for either trust or mistrust (1, 4). When adult patients trust their physician, they feel less vulnerable, physicians feel more effective, and patient-physician communication quality increases (5, 7). When trust does not develop, or is eroded, patients and families can experience anxiety, frustration, second-guessing of key medical decisions, and broken patient-physician relationships, while physicians are unable to provide the optimal care that patients and their families need (6). Finally, in recent decades, where patient trust of both individual physicians and institutions has eroded, lower levels of trust present an impetus to ensure that patients receive clear communication and meaningful inclusion in decisions about their health (5, 7, 8).

In the pediatric healthcare context, trust has important nuances and implications for medical care. Development of trust in the pediatric setting is further complicated by the triadic relationship involving the child, parents/caregivers (herein referred to as parents) and physician (6). Sisk and Baker developed a model of interpersonal trust in pediatrics that posits that families initially trust physicians based on perceptions of competence but that trust increases when the physician demonstrates they are trustworthy through relationship-building (6). Physicians’ actions determine this *relation-based trust* as parents interpret the physicians’ actions in a continuous cycle. Other studies have endorsed the value of ongoing relationships in building and maintaining trust between physicians and parents (9, 10) as well as repairing and re-establishing trust in previously difficult relationships (11). Existing pediatric research has focused mainly on trust in the context of longitudinal outpatient relationships with primary care physicians or specialists (12, 13). Currently, medical literature does not include studies that explore the process of establishing trust between parents and physicians in the pediatric inpatient setting. The inpatient setting is less likely to include longitudinal patient-parent-physician relationships and may increase patients’ and parents’ stress and vulnerability; these setting-specific factors could produce fundamental differences in how trust is built between parents and pediatric physicians.

Children with Medical Complexity (CMC) are one of the fastest-growing inpatient populations in pediatrics (14). They have multiple chronic conditions, high health care utilization, and medical technology dependence (15). Similar to adult patients with chronic disease (16), parents of CMC expect trusting relationships with physicians ideally to be reciprocal, as opposed to the traditional view of the patient or parent as trustor and physician as trustee (2, 13, 17). In the setting of complex and chronic diseases, physicians are often compelled to place trust in the competence of patients/parents and the expertise they bring through their lived experiences. CMC are more likely than other patients to have experienced medical errors and uncertainty in management, including delayed diagnoses (18–20); all of these adverse experiences have the potential to erode trust (21, 22). For parents of CMC, physicians’ medical expertise may be insufficient to inspire implicit trust, since parents of patients with complex and/or rare diagnoses may have more condition-specific knowledge than do physicians (23–26). CMC also tend to experience frequent hospitalizations, which often involve discontinuous relationships with inpatient physicians, which may impact the ability of CMC and their parents to develop trust in physicians (27, 28).

The aim of this study was to investigate how parents of CMC conceptualize trust in the triadic parent-CMC-physician relationship in the inpatient setting. No studies to date have specifically explored the development of trust between parents of CMC and inpatient physicians. The needs and experiences of parents of CMC are unique and important due to their child's frequent hospitalizations, their own child-specific expertise, as well as historical and contextual factors that may prove challenging for trust formation. This study seeks to understand trust development in the inpatient setting from the perspective of parents of CMC, with the goal of enhancing understanding of how to promote trusting relationships between parents and physicians in general.

## 2 Methods

### 2.1 Study design

We used interpretive description methodology to structure this study, which involved collection of firsthand accounts from parents of CMC about the factors that influenced their development of trust in their child's inpatient physicians (29, 30). Interpretive description is an inductive qualitative analytic approach that helps to understand human experiences that are both constructed and contextual in nature. This approach aids in the identification of patterns and themes within data and fosters development of broad understandings that can directly inform clinical practice (30). Another feature of this methodology is a heightened awareness of “outliers” which are perspectives that might otherwise be missed in a thematic analysis if mentioned by only one participant (31).

### 2.2 Setting and recruiting

Parents of CMC who were hospitalized on inpatient care units at the Alberta Children's Hospital (ACH) were recruited to the study. Inpatient care units at the ACH are overnight units that do not include emergency care, intensive care, or oncology. All interviews were conducted in English. Parents of children who met all inclusion criteria for CMC, and were older than six months, were eligible to participate. The definition of CMC was adopted from Complex Care Kids Ontario in Table 1 (full criteria in Supplementary Material S1) (32).

**Table 1:** Adapted complex care kids Ontario standard operational definition.

1	Child is dependent on medical technology at home
2	Child's care requires involvement of at least five healthcare practitioners/teams
3	Child has significant fragility as evidenced by at least two prior hospital admissions and/or at least one admission to the Pediatric Intensive Care Unit

Parents were recruited through a co-occurring prospective Research Ethics Board-approved study investigating the development of post-traumatic stress symptoms (PTSS) in parents of CMC (hereafter referred to as the PTSS Study). This study was conducted in an inpatient setting. Only parents who had completed the PTSS Study were recruited. (See Supplementary Material S2 for recruitment process for PTSS study). Parents’ responses demonstrated a range of scores on survey instruments completed for the PTSS Study including the Post-traumatic Stress Checklist and the Pediatric Trust in Physician Scale, reflecting a broad range of stress, trauma and trust levels. The scores on these surveys for the PTSS study were not a condition for inclusion in the Trust Study nor were they included in any analysis for the Trust Study. Forty-three parents consented to be contacted for future research and were invited to participate in this study (hereafter referred to as the Trust Study). Written consent was obtained at enrollment.

### 2.3 Data collection

Two senior researchers conducted one-on-one semi-structured virtual interviews with participants that lasted 45–60 min, scheduled between June 2022 and August 2023. Interviews for the Trust Study took place between 68 and 323 days after the CMC patient discharge date from the original PTSS Study. Using secure Zoom videoconferencing, interviews were audio-recorded and professionally transcribed and redacted for identifying information.

A semi-structured interview guide was used for questioning (see Supplementary Materials S3). Participants were asked about: (a) their child's most recent hospitalization (where they enrolled in the PTSS study) and their level of trust with the physicians they encountered during that hospitalization; (b) previous positive and negative trust experiences when their child had been hospitalized prior to the most recent hospitalization; (c) advice they would give to physicians and also to other parents about building trust; (d) their reflections about whether physicians trust them.

The interviews approached the topic of inpatient physician trust with few preconceived notions, encouraging participants to talk about their views and experiences openly. “Inpatient physician” referred to any physician who was involved in the child's medical care while hospitalized, including post-graduate trainees (residents or fellows). While the interviewer asked participants to focus on their experiences when their child was hospitalized and comment on trust of inpatient physicians, participants sometimes related stories about community or emergency room physicians, or nurses, to give further examples or contextualize discussion. These comments were also incorporated into the thematic analysis.

Each senior researcher conducting the interviews was aware of their positionality, which refers to the identity of the researcher in relation to the participant, including personal characteristics such as gender and socioeconomic status as well as past experiences and context (33). Researchers reflected on potential biases that could affect data collection or interpretation. Both interviewers had experience conducting interviews with individuals who have medical complexity and/or disability. Interviewers attempted to make participants feel comfortable despite potential perceived power or knowledge differentials. The tone of these interviews was conversational to encourage participants to feel comfortable and open up about their parenting experiences and thoughts about trust. Interviewers incorporated breaks when needed and offered supportive and sympathetic words if participants became emotional. Participants were also reminded that they could choose to leave the study at any time without the need for explanation and could skip any questions that they felt uncomfortable answering.

Recruitment ended when all eligible parents had received an email invitation and reminder to participate. Interpretive description methodology cautions that assumptions about theoretical saturation should be made carefully. New perspectives and ideas could always arise with more data gathering; however, the research team felt confident that many of the same themes were being discussed in later interviews thus indicating a reasonable consistency within the accounts had been reached (29, 34).

### 2.4 Data analysis

Two senior researchers conducted the interviews and, along with the study's primary investigator (PI), created research memos from each interview. Analytic research memoing is a process of making sense and refining thoughts that develop into ideas as the researcher encounters the data (30). Memoing facilitates the organization of data, visualization of important connections, and conceptualization of themes (30, 30). A core analysis team, including the principal investigator, two senior researchers (who conducted the interviews), and a parent-partner then debriefed the interviews and memos. Creating research memos and continuously reflecting on the data coding process allowed researchers to develop comprehensive thematic groupings that stayed consistent with the participants’ data. Core analysis team members also brought different perspectives to these debriefing discussions, including clinical and personal experiences, that provided deeper understanding and guarded against researcher bias. Interpretive description methodology encourages researchers to practice reflexivity—researcher self-awareness and self-evaluation—during the research process. This practice encourages researchers to consciously examine their own biases and perspectives to prevent these biases from unduly influencing the research process (35).

A senior researcher conducted in-depth coding of all interviews with NVivo software. Two researchers inductively developed the initial coding scheme using the first five interviews. One researcher then completed the coding and continued to refine and apply the coding structure to the remaining transcripts. The core analysis team regularly reviewed the coding process as it evolved. This detailed inductive coding process allowed researchers to see a developing list of themes based on positive and negative practices that participants felt could help promote or inhibit triadic trust relationships with physicians (see Supplementary Materials S4).

All study participants were invited to participate in a member checking process where they were asked for feedback on a preliminary description of the themes. Five participants responded to the request with two supporting the thematic analysis as presented, and three providing further thoughtful comments that were incorporated into the final interpretive description analysis.

## 3 Results

### 3.1 Participant characteristics

Sixteen parents of unique patients consented to participate in the study with an acceptance rate of 37%. Each participant also completed a short demographic survey before the interview. Thirteen female (81.25%) and three male (18.75%) parents of CMC (herein “parents” or “participants”) were interviewed with an average age of 39 years and a range of 26–57 years old. Table 2 summarizes the demographic features of participants.

**Table 2:** Characteristics of participating parents of CMC.

Category		*N* (%)
Education		
	Some high school	1 (6.25%)
	Some college/university	2 (12.5%)
	Completed technical/trade schooling	3 (18.75%)
	Completed university degree	7 (43.75%)
	Post-graduate education	3 (18.75%)
Household income^a^		
	$30,000–49,999	1 (6.25%)
	$50,000–69,999	1 (6.25%)
	$70,000–99,999	2 (12.5%)
	$100,000–149,000	6 (37.5%)
	$150,000 or more	3 (18.75%)
	Prefer not to say	3 (18.75%)
Relationship status		
	Married/common law	14 (87.5%)
	Divorced	2 (12.5%)
Ethnic/racial background		
	White/European	11 (68.75%)
	Southeast Asian	2 (12.5%)
	Indigenous	1 (6.25%)
	Latin American	1 (6.25%)
	Other—mixed race	1 (6.25%)

^a^ Canadian dollars.

### 3.2 Meta-theme: need for situational awareness and personalization of care

The themes derived from the multi-step analysis illustrate how to create a foundation for trust from the perspectives of parents of CMC. Factors that influence parent-physician trust span many important aspects of the physician-parent-child encounter and relationship. Trust is a fluid feature in relationships—it develops on a continuum that can deepen and strengthen with positive encounters or weaken or break with negative ones. Every parent was at a different stage of how trusting they could be with an inpatient physician or physicians in general. Throughout the interviews, parents spoke about an overarching theme: the need for situational awareness and personalization in building trust.

Evident as a running theme across of all the interviews was the need for physicians to have “situational awareness” to gain the trust of parents of CMC. Situational awareness can be viewed as a deliberate conscious knowledge of the different elements and circumstances in a clinical situation (36). From parents’ perspectives, this knowledge and understanding related to the child, the parent, their collective history, and the context of the clinical situation. To facilitate trust in these often highly challenging encounters, parents felt this knowledge must then be translated into a personalization of the physician's approach.

Pertaining to situational awareness, parents expected physicians to appreciate the unique, and often rare, nature of their child's condition. When parents were concerned that physicians were failing to incorporate this child-specific knowledge into their clinical decisions, this concern impeded the formation of trust.

“I think a lot of the failures that we've had is because it was a cookie cutter approach, applied to a situation that was way more complex. If you were just to breeze by it, you'd be like, ‘Oh, it’s just this.’ But if you actually took the time and you asked the right questions, you would not have applied a cookie cutter approach” (P11).

Some parents also pointed out that physicians need to tailor their interactions to children of different ages and stages, especially older or more mature children, to gain their trust.

“And what was better for [child name] based on her style, which is slow and steady, let her think about things. […] And if you say to her, ‘Well, should we do this today?’ Her first answer is, ‘No.’ So Dr. A figured her out pretty quick and asked what’s the best way to deal with her” (P7).

Parents of CMC had varied needs and desires relating to physicians, managing the healthcare encounter, and building trust. Although all parents wanted the focus to be directed to their child, some appreciated attention and acknowledgement from inpatient physicians relating to their own health, coping, and contributions.

“They'd ask about how work was or whatever […] just taking that extra little bit of time and discussing something else […] I mean, your brain, you're running on little to no sleep for periods of time. It’s not your best self, right there. So yeah, when they ask that, it definitely helps reduce stress. And it’s not just talking about your sick kid the whole time” (P5).

Some parents reported a need for inpatient physicians to understand the context of the clinical encounter to enable trust-building. Many parents recognized that they were often “not at their best” during these hospital admissions. They desired inpatient physicians to appreciate the effects of stress, burnout, sleep deprivation, and fear on their interactions. Other parents preferred to have the physician focus completely on their child's needs indicating that not all parents expected to have an emotional connection with the inpatient physician or care team.

“I don't tend to need emotional counseling from a physician just because that’s not what they're there for. That’s not what I need them for. If I'm upset about [child name], I have my wife, I have other supports that I'll rely on” (P6).

There was variability in how parents of CMC felt that communication between parent and physicians ought to take place. For example, some parents in this study acknowledged their own expertise as “medical moms and dads” and felt that they should be treated differently than parents of non-CMC.

“I had never met him before this. So yeah, he kind of picked up right away that I was a more medical mom and that he could use more jargon with me and that he could speak more freely. I've not seen him talk to other patients, obviously, but I liked that” (P9).

Many parents in this study felt that inpatient physicians’ ability to tailor communication to meet their needs and preferences was integral to building trust and supporting the parent-physician relationship. One parent related how a simple but effective communication tool was helpful in promoting trust for her:

“I really liked […] the board on the wall, which has opportunities for parents to write things. And doctors will come in, they'll put their name for the day, here’s our goal, here’s what we need to accomplish today […] and sees, ‘Oh, this parent has left me something. They've got a question.’ Or they said, ‘Hey, this thing needs to still be done. Don't forget.’ I think that those are also ways to show, ‘I trust you by sharing this knowledge and hopefully you trust me back by sharing more knowledge’” (P16).

Many parents of CMC revealed how specific past experiences—particularly negative experiences and trauma—influenced their ability to be trusting. Events such as medical errors, missed diagnoses, and hurtful or unsafe past experiences clearly hindered trust development. Some parents also referenced these past events to justify their own approaches and behaviours in the clinical encounter.

“To be perfectly honest, ever since [child name]’s been born, doctors have messed things up. So, I always have that underlying. I'm always very clear and I repeat things several times and I'm always asking questions. I'm not like that chill mom, that’s just like, ‘Oh, they're going to do their job.’ I feel like I have to be on it all the time. […] Because there’s been a lot of things that have been missed over the past two years” (P11).

A few parents felt obligated to never leave their child's side during hospitalization because of previous medical errors, concern for their child, or perceived neglect of their child.

Finally, some parents of CMC reported the importance of developing their own situational awareness about the hospital environment, including system-specific factors such as the constraints of the hospital environment and inpatient physicians’ working conditions, as well as more person-specific factors such as inpatient physicians’ individual stressors and personality traits. During the interviews, several parents reflected about their own role and behaviour at the hospital. One parent suggested treating the hospital “like a workplace” by showing respect for staff and for the hospital environment (P9); another parent acknowledged that the health care providers caring for children are also under a lot of stress.

“We treat our team as humans, not just as somebody there in a medical role. You know, we make sure that we're really conscious to pay attention to names…you know, to speak, and to talk kindly, to listen too, and remember that every single one of our medical team has a family or a partner that they go home to every night” (P10).

### 3.3 Summary of themes

In addition to the overarching theme for the need for situational awareness and personalization of care, responses from parents evoked six themes that relate to trust development, which are outlined in Table 3 along with illustrative quotations.

**Table 3:** Summary of themes related to trust development.

Theme	Description	Trust facilitators	Trust barriers	Quotations
Ensuring that the focus is on the child and family	Putting the child's needs as the focus created the foundation for a trusting relationship. Acknowledge and address parents’ needs during hospital admissions. Prioritize building a connection and relationship with the family.	• Expedite or facilitate a child's treatment. • Be proactive about the holistic needs of the child • Advocate for treatments on behalf of the child. • Ask parents for permission to perform a procedure. • Trust parents to care for child at home. • Take time to check-in on parents’ well-being.	• In a rush • Ignore child • Ignore parent(s) • Lack of empathy	*Yeah, or if they talk to my daughter, a lot of them don't do that. They just talk to me, so I like it when they interact with her, because she's the actual patient. Obviously, they talk to her when they’re doing the exam, or whatever. Some of them give her stickers, which is nice because I think they sometimes forget that, obviously when she was little it was different, but now I feel she understands certain things that are happening with her* (P8). [T]hey asked us almost every time we saw them, “How are you guys doing today? Do you want the full thing? Or do you want the Coles Notes version?” […] I mean, your brain, you're running on little to no sleep for periods of time. It's not your best self, right there. So yeah, when they ask that it definitely helps reduce stress. And it's not just all about talking about your sick kid the whole time (P5). *I'm burnt out and I've fallen between the cracks. So, if we're not going to handle it with a parent telling you, “I'm not okay,” then…And when you tell that emerge doc that I'm not okay, and she says, “Well, I can't refer or do anything,” okay, well, we're in trouble. You're going to escalate the problem* (P12).
Respecting both parent and physician expertise	Parents want physicians to have medical expertise, but also trust more easily when physicians are honest about gaps in their knowledge. Parents want acknowledgement for their own expertise, helps them to trust easier.	• Have familiarity with their child's medical history. • Ask parents for their version of the child's problem. • Be honest about gaps in knowledge or uncertainty. • Acknowledge and respect the expertise that parents bring.	• Dismiss parents’ concerns. • Create power differential.	*We didn't really have a good experience with the physician there for trust. […] Just with the lack of understanding of CHILD's condition, I didn't really fully trust them to make the right decisions* (P14). Really, I don't see how they can have all the information for everything all together, all the time in their head. And my child is different enough that she has defied medical everything right from the beginning. So, the fact that a doctor can turn around and feel comfortable enough to admit that they don't know something is, for me, hugely important (P1). *So, we do feel heard when we bring things up to her, when we bring crazy ideas up to her, knowing that she has more medical experience than we do, but we have more CHILD experience than she does* (P1). I think I operate in terms of medicine at a relatively high level. I'm often asked if I work in healthcare. Some people even ask if I'm a doctor. You just pick things up as a parent of a “frequent flyer”. And the best relationships with physicians [are] generally when they recognize that, and they respond in a way that just cuts to the point without, I guess, the fluff that's required to communicate more with lay people (P6).
Collaborating effectively	Parents want physicians to collaborate with them, their child, and other team members. This also includes negotiating roles that are fair and reasonable. Some parents felt that they also need to recognize the rules of the “medical space” to collaborate effectively.	• Involve parents as a member of the care team, • Support parents in their decision-making. • Communicate and collaborate with the whole care team. • Explicitly delineate roles of parents and staff when children are in hospital.	• Lack collaboration with family. • Lack collaboration with care team e.g., make changes to care plan unilaterally • Fail to follow up with family when promised or when results are ready.	*I feel like I can trust them when I get the sense that this is a collaborative relationship and we're all here to take care of my son […] instead of the parent being outside and then the team over here trying to figure things out* (P2). We've had situations where the resident comes in and checks in the morning and then the attending comes later and it's like they haven't talked to each other all day and then you're just repeating yourself. […] You lose a little bit of trust because sometimes you're like, “Are you guys even working together?” (P5). “*I didn't ever feel like nurses or doctors didn't trust me. I felt the opposite like they expected more of me than what should be. I've often felt like I'm a volunteer on their medical team expected to assist them during admissions”* (P11) I don't know how to fix these machines, run these machines, do all these things. So, I think that there is a way to show a doctor that you are a parent that can be trusted by respecting that those are their tools […] and knowing that I'm going to approach this delicately and ask for help and ask for advice before I start doing something. And that just allows us to share that space commonly in the most peaceful way possible (P16).
Maintaining a flow of communication between parents and physicians	Parents wanted physicians to listen actively and carefully, accommodate parents’ needs and preferences regarding style of communication and level of detail. Parents felt that asking and answering questions was critical to the development of trust, to feel that their concerns are not being dismissed.	• Physician needs to introduce themselves, be open to questions and provide clear, concise answers. • Active listening skills. • Tailor communication and information sharing to the needs of the parent. • Thorough explanations and information.	• Sugar coating reality of situation • Insensitive communication e.g., sharing bad news • Using language not appropriate for family e.g., medical terminology or phraseology (note that some CMC families want physician to use technical language as this reflects parent's competence/understanding)	*“If they*'*re just not listening, not letting us explain, not taking the time to ask questions and just stating that, “No, this is the way it is,” then yeah, I'm not going to trust this physician at all” (P13)*. Really advocate for your child. And also, yourself, be personable. If you go in with guns blazing, assuming that nobody's going to listen to you, and getting excited and whatnot, that's probably not the best way to…the doctor's probably going to get their guard up and whatnot. So, calmly explain the background, what has happened in the past, what has worked for treatment in the past kind of thing, to help the doctor do their job, I guess, to give them all the information that they need (P13). *He doesn't just tell us, “Oh. We should do the ketogenic diet and that's that.” He's like, “I want you to learn about the ketogenic diet and tell me what you think” (P1)*. We also get copies of pretty much all of his testing. […] I have to see the results myself which I'm sure most parents probably don't and it's probably because I am a healthcare professional, but I need see it in writing because ever since the NICU, we had things missed that then I later found and needed follow-up on (P2). *[W]hen the teams come through, it doesn't matter what they're talking about, if we have a question, they'll sit down and they'll stop what they're doing, and they'll break it down for you, and they'll give you an explanation, and then they'll continue. I've always liked that. And they don't make you feel stupid for asking a question, which sometimes, you know, is hard to do, especially when you're dealing with a bunch of really smart people in a room (P15)*.
Acknowledging the impact of personal attributes	Parents described positive and negative characteristics in physicians that encouraged or discouraged trust building. Parents also acknowledged that sometimes personalities clash and tried to rise above these differences.	• Traits that promote trust for parents include honesty, respect, compassion, friendliness, a sense of humour and open-mindedness.	• Other characteristics damaged trust such as being cold, confrontational, clinical, distracted, or in a rush.	*She's so kind. I just love her so much, this pediatrician, she's done everything for us* (P9). I wouldn't know if there's necessarily a breach of trust, but I find the ones that don't have great bedside manner are hard to trust if they're really cold or I feel like some of them maybe forget that we're human and that they just go about it like it's their job (P8). *So I just try to focus on the fact that they have experience in the field for a reason and not everyone has the right people skills and that I can kind of put that aside* (P2).
Recognizing issues related to the health care system	Includes discussions of the healthcare system and other issues that contributed to parents’ ability to trust. Parents expressed frustration with systemic issues they felt powerless to change. Some parents realized physicians were not always at fault for their loss of trust, but rather perceived flaws in how the system delivers care and were thankful for caring individuals.	• Continuity of care • Facilitated transfers • Expedited treatment • Effective crisis management	• Miscommunication between depts • Staffing issues or shortages • Long wait times • Lack of supplies • Medical mistakes • Patient transfers • Poor crisis management	*Trust is not about the individual to me, obviously in the context of healthcare. It's about how the care will be provided. When I don't trust the system, yeah, that's a good way to say I don't trust the system right now. It's not that I don't trust that any individual nurse wants to do the best job that they can, or any individual physician isn't working their ass off. It's just that the result won't be good care unfortunately right now* (P6). I wish there was a way that the nurse, when you're giving the nurse the information, she's able to triage and understand. […] But sitting there for an hour and a half waiting for a doc to write a requisition is painful when the [requisition] takes no time, and why can't we take into consideration that this is a complex kid with a DNR and a hypoplastic left heart? If this kid sits in emergency and gets a cold […]…Why can't we be thinking of him? (P12). *With CHILD's care, there have been many mistakes that have been made from meta errors, which I think happens to many people who spend any kind of time in hospital. We've had formula that was made wrong, because again, there was a communication error […] And again, it exposed a pothole or a sinkhole or whatever, to better care and better plan for things in the future so other children don't go through it, you know?* *And that there's a lot that I feel CHILD has exposed at the HOSPITAL of, “We need a better way to do this” (P1)*. Just the kindness of the nurses at the children's hospital. Once we were admitted, they were really kind and really empathetic towards what we were going through, and they really did everything they could to make our stay welcoming, almost, if that makes any sense (P14).

#### 3.3.1 Theme #1: ensuring that the focus is on the child and family

All parents in the study wanted their child's needs to be the central focus during hospitalization. In addition, many parents felt that forming trust also required that physicians (whether inpatient or outpatient physicians) acknowledged and gained understanding of the needs and experiences of parents themselves. However, not all parents of CMC felt that they needed this acknowledgement or attention from inpatient physicians to develop trust. This approach, focused on the child and family, was supported when physicians prioritized building a connection, an undertaking that was made easier if there was continuity of care. Parents valued continuous relationships with physicians across encounters as this continuity facilitated awareness, familiarity and understanding of the child and parent.

#### 3.3.2 Theme #2: respecting both parent and physician expertise

In the therapeutic encounter, physicians bring medical expertise and parents bring child-specific expertise. Parents feel that both need to be respected in order for trust to develop. Parents described how they had a baseline level of trust for all physicians given their medical knowledge and training, but this was sometimes called into question when inpatient physicians lacked knowledge about their child's complex and/or rare condition. In other instances, medical mistakes or disagreements over treatment plans also caused parents to doubt physician knowledge and expertise. Paradoxically, many participants felt they trusted more easily when a physician was honest about their level of knowledge or admitted gaps in their understanding. Parents also brought considerable hard-won expertise from their own inquiry, day-to-day care of their child, and repeated hospitalizations. Some parents felt that this intimate and experience-based understanding of their child made them different from parents who do not have CMC. Every participant reported that when physicians acknowledged and respected parents’ expertise about their child, they were able to feel more trusting towards inpatient physicians.

#### 3.3.3 Theme #3: collaborating effectively

Parents indicated that the ability to collaborate effectively was another key element for building trust with inpatient physicians. When physicians took an inclusive approach to providing medical care with emphasis on shared decision-making with parents of CMC (as well as CMC themselves when maturity and cognitive ability allowed), parents felt that their expertise was validated by physicians and that this practice supported trust-building. However, some parents pointed out that the stress of decision-making and negotiating the various roles needed to care for a child in hospital can cause friction or misunderstanding; effective collaboration must include clarifying these roles and expectations between parents of CMC and an interdisciplinary medical team. Some parents felt that the medical teams’ expectations of them were excessive, unreasonable or unsupportive. Parents also expected physicians to collaborate effectively with others (including trainees, nurses and other physicians); when this collaboration did not happen, trust was negatively impacted. One participant pointed out that parents also need to be respectful and cognizant of medical spaces that have their own rules, to be able to collaborate effectively with the child's medical team.

#### 3.3.4 Theme #4: maintaining a flow of communication

Another consistent message from parents was that maintaining a flow of communication is central to developing trust with inpatient physicians. Many participants felt that active listening skills were critical as parents had experienced not being *heard* when their child was hospitalized. Parents appreciated when physicians accommodated parents’ preferred communication style (e.g., written, verbal, and diagrammatic) and need for information. Many parents spoke about the importance of being able to ask questions of their child's inpatient physicians and their appreciation of being invited to do so. When physicians encouraged parents to ask questions, parents believed that their concerns would be thoughtfully addressed. Parents also pointed out that non-verbal cues, such as sitting down as a signal of not being in a rush, can promote trust. Some participants acknowledged that how *parents* communicate is also very important, can affect how successful they will be in advocating for their child, and can influence whether inpatient physicians trust parents of CMC.

#### 3.3.5 Theme #5: acknowledging the impact of personal attributes

Parents of CMC recognized the impact of personal attributes and felt that building trust was easier with inpatient physicians who had characteristics such as honesty, compassion, open-mindedness, friendliness, and humor. Sharing personal stories also helped to “humanize” physicians, allowing parents to trust these physicians more easily. Parents also identified characteristics that discouraged the formation of trust and described these inpatient physicians as “cold,” “distracted,” or “rushed,” or as having a more “clinical” approach.” Parents acknowledged that sometimes personalities clash and that “no one is perfect.” Some parents were understanding that inpatient physicians experience stressors that can test their ability to be positive or kind. These parents were willing to accept that inpatient physicians are “doing their best” and felt that this attitude was helpful in trust-formation.

Many parents in the study reflected on the impact of their own personal traits on the trust-building process. For example, parents of CMC exhibited variable levels of confidence in terms of how they navigate hospitalizations with their child. Confidence affected whether a parent asked for a second opinion, reported disrespectful treatment from a physician, or could recover from a broken trust experience. Some parents were fiercely proud of being tough “medical moms” who kept meticulous track of their child's medical history and had lived through medical trauma. Other parents were worried that their stress responses could result in them being labeled as difficult, using words like “crazy” to describe themselves. Finally, several parents pointed out the importance of being “personable” and calm when advocating for their child.

#### 3.3.6 Theme #6: recognizing issues related to the healthcare system

Parents of CMC asserted that issues related to the healthcare system affected trust development in the inpatient setting, with some factors being conducive to trust-building and others having a detrimental effect on this process. Systemic shortcomings such as staffing issues, lack of equipment, suboptimal crisis management (e.g., during Covid-19 pandemic), and inefficient interactions between departments (e.g., when patients are transferred from one unit to another) were the kinds of issues that resulted in a loss of trust in the “system” and those employed within it. Some parents pointed out gaps in family and parent services in the pediatric healthcare system (e.g., lack of availability of parent mental health support during hospitalization) and how these shortcomings could also decrease trust in the system. Parents also recognized that many of the systemic issues that affect parent-physician trust are difficult to fix. Although many participants shared their frustrations with the healthcare system, they also offered suggestions for how to improve it.

## 4 Discussion

### 4.1 Trust as an outcome of patient- and family-centered care

This study revealed numerous themes that are critical to relationship-building and trust-building between parents of CMC and their child's inpatient physicians. Parents felt that parent-physician trust-building in the inpatient setting required that the physician demonstrate a clear focus on the child and family as well as good communication skills. They also desired respect for their own expertise alongside the respect they felt for the physician's knowledge and experience. Collaboration between parents and all members of the inpatient healthcare team, evidenced by mutual respect and role negotiation, helped establish parental trust in inpatient physicians. Parents appreciated that certain characteristics and behaviours of themselves and inpatient physicians could facilitate or hinder the development of trust; understanding these differences allowed some parents to tolerate some traits and behaviors that otherwise might have impaired the relationship. Finally, systemic factors also influenced the trust that developed between parents and physicians.

These elements of trust-building are reflected in other pediatric populations and settings. Notably, a study by De Lemos et al. (27) also emphasized several parent-identified trust facilitators, including physician communication (clarity and frequency), collaboration both with parents and within the healthcare team, and parental involvement in decision-making (27). However, in that study's broad population of children with acute and chronic conditions, the need to integrate parent and physician expertise and to develop situational awareness were not identified as important facilitators. In a study on one subpopulation of CMC, children with Trisomy 13 and Trisomy 18 (also CMC), Janvier et al. discuss the need for personalization (23). In Janvier et al's study, personalization of information to the child and respecting different decision-making preferences was a key theme in the facilitation of trust. Although not specific to trust, a study in a pediatric oncology population (11) identified problems with connection and understanding as a core issue in difficult therapeutic relationships with low levels of trust.

Many of these themes align strongly with the principles of patient- and family-centered care (PFCC). PFCC is an approach to the planning, delivery and evaluation of healthcare that is grounded in collaboration and partnership with patients, families, and healthcare providers, recognizing the centrality of family in a child's life (37). The core concepts of PFCC that matched the major themes of trust-building that we identified in this study include: (a) Respect and Dignity (listening to patient and family perspectives and incorporating these perspectives into management); (b) Information sharing (sharing information that is timely and accurate); (c) Participation (shared decision-making); and (d) Collaboration (among patients, families, healthcare providers and leaders) (38, 39). Since adherence to these principles is endorsed as a means of facilitating the provision of high-quality care, it is not surprising that parents also identified these as critical to developing trust with physicians. Our findings reinforce and expand upon the value of the PFCC principles by emphasizing their role in developing trusting relationships between parents of CMC and inpatient physicians. Trust, one might say, is an outcome of applying PFCC.

Parents of CMC hold essential knowledge and expertise in their child's condition, their history, and unique insights into the care of their child. Learned via day-to-day experience, this parental expertise is inherently different from that of physicians and is especially valuable to the medical care of their child. This child-specific knowledge allows parents to collaborate at a different level than parents who are naïve to the healthcare system or whose children have conditions with clear diagnoses and management guidelines. At the same time, this personal experience often includes traumatic experiences in healthcare, including occasions of broken trust and medical errors, which may impact their ability to form trust in future therapeutic relationships. Several parents shared how many aspects of caring for their child can also be very traumatizing, such as performing painful medical procedures (P9), witnessing painful procedures when their child is hospitalized (P1, P2, P4, P5, P13), dealing with unexpected diagnoses (all participants), or facing life-or-death decisions about their child (P1, P3, P4, P13). Other studies have also shown that this intensity of involvement in the inpatient setting comes with high levels of pressure and responsibility for parents (40, 41). Considering that CMC can be amongst the most challenging of patient populations (42), it follows that families of CMC might also be most in need of PFCC. Inpatient physicians can better treat CMC and support their parents by navigating the line between necessary parental involvement and overwhelming parental responsibility when their child is hospitalized.

Many examples from the research interviews in this study illustrate how parents of CMC highly value the concepts inherent to PFCC. The needs of CMC patients and families also illustrate the benefits of PFCC. For example, information needs to not just be “shared”; rather communication needs to be precise, considerate, and transparent. For CMC and their parents, patient- and family-centered communication requires that physicians know how best to interact with a child with severe developmental delays, to prepare children and families emotionally for difficult or painful procedures, and to discuss decisions with life-and-death implications respectfully with CMC and their parents. Combining the concept of PFCC with an individualized care approach ensures that inpatient physicians do not make assumptions about parents of CMCs’ skills and feelings. Providing PFCC improves the likelihood that all patients and families receive individualized management in an environment that facilitates trust-building with their physicians.

### 4.2 Critical trust and the guarded alliance

One could posit that building trust between physicians and CMC and their parents is an undisputed requirement for optimal management of medical problems. Even so, for parents of CMC to have implicit trust in inpatient physicians may not be in the child's best interest. In this study, most parents of CMC approached trust as something to be earned and reciprocated. Parents were often wary of relationships with new physicians, whether because of past negative experiences or their need to be vigilant about their child's medical care. Parents of CMC also operate within a system where lower levels of trust and higher expectations for patient-centered care are increasingly the norm (7, 8). Thorne and Robinson coined the term “guarded alliance” to describe the final stage of trust development amongst patients with chronic illness, their families, and healthcare providers (43). In their study on adults with chronic illness, Thorne and Robinson described three stages of how trust evolves over time: naïve trust, disenchantment, and guarded alliance (43). In the last stage, patients had various reactions to their positive or negative healthcare experiences that shaped their ability to trust—ranging from hero worship, to resignation, to consumerism, to team playing. The latter, most beneficial outcome is a healthcare provider-patient relationship based on *reciprocal* trust (16). Parents of CMC also exhibited many different stages of trust. Similar to Thorne and Robinson's paper, most parents felt that their definition of trust required both parties—inpatient physicians and parents of CMC—to work towards establishing a trusting therapeutic relationship. Parents of CMC felt that inpatient physicians that take the time to understand parents’ past experiences, acknowledge parents’ struggles, and trust parents’ perspectives demonstrate their trustworthiness by showing care and respect for parents. Parents of CMC who can acknowledge (and forgive) inpatient physician fallibility were better at building trust and demonstrated a sense of self-awareness regarding whether they were trustworthy themselves.

### 4.3 Personalizing care to build trust

Importantly, parents of CMC did not have identical perspectives on trust-facilitating approaches which is captured in the overarching meta-theme of situational awareness and personalization of care. The concept of “personalized care” is complementary but distinct to that of “personalized medicine.” The latter focuses on screening, prevention, and treatment plans that are tailored to an individual's biological or environmental risk factors (44). In contrast, personalization of care (45, 46) reflects the control patients can exert over the way their care is delivered. This concept overlaps with the tenets of PFCC (incorporation of the patient's and family's preferences and values) and culturally competent care (consideration of the patient's and family's culture and preferred language). For the parents of CMC in this study, the concept of personalized care was expanded to include situational awareness, which incorporated historical, situational, and systemic considerations. In essence, situational awareness in the clinical encounter implies gathering thorough information about the child and their family, using this information to interpret or predict their needs, and incorporating this information into the health care provider's approach (47).

One novel concept that we uncovered through this study was that personalization of care involves not only incorporating patient values into a medical decision or plan of treatment but also *adapting the therapeutic relationship itself* to meet the individual needs of patients and families. This practice can be challenging for physicians, who are often taught standardized and translatable skills that can be used across clinical encounters (48, 49). Although training in specific skills undoubtedly has an important place in the practice of medicine, a standardized and rigid approach is likely not sufficient for working with CMC and their parents. Instead of a “best practice” for building trust with CMC and their parents, physicians must assume a position of flexibility and incorporate an individualized approach for each unique patient and family. By incorporating awareness of critical elements (such as patients’ and parents’ preferences for support and communication, and recognition of traumatic stress related to previous experiences in medicine), physicians have the best chance of achieving trust and the guarded alliance.

## 5 Limitations

Convenience sampling recruited participants from a separate study addressing post-traumatic stress symptoms (PTSS) in parents of CMC. Participants in the PTSS Study were not limited to those who had experienced PTSS. Thus, this recruitment strategy was not expected to significantly influence the outcomes of this study. Within a qualitative study, we cannot account for differences in important demographic and clinical characteristics among participants (such as child age, number of previous admissions and language preferences). As such, these results may not be generalizable to all hospitalized CMC. Significant inter-relatedness in content was found between interviews, but these data still may not represent the full breadth of experience of the diverse population of hospitalized CMC and their parents. The study sample was also not demographically diverse as participants were mostly white, wealthy, female, married, English-speaking and Canadian. Although the interviews asked directly about experiences with inpatient physicians, some participants shared experiences from other settings and with other types of professionals. For example, participants mentioned interactions in the emergency department or with a medical trainee or nurse. Although these topics are outside the scope of this study, we felt it was important to allow participants to share these events, which were obviously very significant to them. These comments were not explicitly excluded from our analysis and could thereby have influenced our results.

## 6 Conclusion

This study provided a qualitative examination of trust development between parents of CMC and inpatient physicians using a methodology that focused on practical applications and translation into “real world” clinical encounters. Physicians require sophisticated skills in order to tailor their clinical approach to meet the needs of each CMC and their family; the physician must be able to personalize their communication style, decision-making, and support so that it is appropriate for each unique scenario. Directed training for physicians and medical trainees would include how to implement trust-building behaviours, suggested by study participants, within the six themes related to trust development (Table 3) such as expediting treatment for CMC, taking time to check on parents’ well-being, being honest about gaps in knowledge, delineating care roles between parents of CMC and medical team, tailoring communication for parents of CMC, and managing crises promptly. This study raised several issues that warrant further study including the challenge of mending broken trust, which represents a particularly noteworthy opportunity for improvement given that every parent of CMC in this study reported a previous instance of compromised trust. In their model of interpersonal trust in pediatrics, Sisk and Baker point out that rebuilding broken trust is difficult and uncomfortable for physicians, requiring skills in conflict resolution and specialized communications training (6). A second phase of this study is ongoing and is focused on physicians’ perspectives, which may provide further insight into the strategies that facilitate the development of physician-patient-parent trust. Triangulation of this study with the physician study will support the development of a toolkit that physicians can use as they work to build trust with CMC and their parents.

## Data Availability

The raw data supporting the conclusions of this article will be made available by the authors, without undue reservation.
