# Supplementary material for: Trust of inpatient physicians among parents of children with medical complexity: a qualitative study
Source: Front Pediatr. 2024 Sep 27;12:1443869. doi: 10.3389/fped.2024.1443869 (PMC11466756; doi:10.3389/fped.2024.1443869)
Supplement: Supplementary file 1 [file Datasheet1.pdf]

Supplementary Material 1

Forming and Maintaining Trust between Physicians, Children with Medical Complexity, and their Parents:

A Qualitative Study of Parent Perspectives

Tammie Dewan,[1] Andrea Whiteley,[1] Lyndsay Jerusha MacKay,[2] Rachel Martens,[3] Melanie Noel,[4] Chantelle Barnard[1], Isabel Jordan,[5] Anne Janvier,[6] Sally Thorne[7]

*** Correspondence:** Corresponding Author: email@uni.edu

Semi-Structured Parental Caregiver Interview Guide

Note: Specific questions may be modified depending on participant responses and feedback from earlier interviews.

Introduction

Thank you for participating in this study. We are hoping to better understand how parental caregivers of children with medical complexity develop trust or mistrust with their child's physicians.

For the interview, I will ask you to tell me about your experiences with developing trust or mistrust with physicians. Specifically, I would like to discuss **those physicians who care for your child during hospital admissions** including this most recent admission where you were recruited for this study. I expect that our conversation will take about an hour.

Do you have any questions before we begin?

Questions

1. Can you please tell me a little bit about your child and your experiences as his/her parental caregiver?

2. Your child (child's name) was recently admitted to hospital. Can you tell me about your experiences during that admission?

3. Who did you trust during that admission and what made them trustworthy?

Prompt: Can you tell me three specific things they did that helped you to trust them? Things they said, things they did, ways they acted.

4. Can you tell me about other physicians in the past that you were able to trust, particularly those who were involved a hospital admission for your child?
   Prompt: What about physicians you did not know well or know at all before your child came into hospital? What specific things did that doctor do or say that helped you build trust with them?
   Prompt: Can you tell me 2 things that physicians can do or say that increases how much you trust them?

5. In this recent hospital admission can you tell me about a physician that you did not trust?
   Prompt: Can you tell me three specific things they did that made it difficult to trust them? Things they said, things they did, ways they acted.

6. Have you had previous experiences where you lost trust or didn't trust a physician caring for your child in the hospital?
   Prompt: Can you tell me about these experiences? What specific things did that doctor do or say that made it difficult to trust them? What was different about these experiences compared to the ones where it was easier to trust?

7. What advice would you give to physicians about how to earn parental caregivers trust during a child's hospitalization, particularly when they don't know your family well?
   Prompts: Are there any actions or behaviours from physician that you think would help? Are there any ways that the way care is delivered that could help trust develop?

8. Have you ever thought about how much your physician trusts you?
   Prompts: Do you think that your child's physicians trust you? How do you know when a physician trusts you? How would you know if a physician did not trust you?
   Prompt: Can you tell me 2 things that parental caregivers can do or say that *increases* how much their physician trusts them?
   Prompt: Can you tell me 2 things that parental caregivers can do or say that *decreases* how much their physician trusts them?

9. What do you think might help physicians place more trust in parental caregivers?

10. If you were able to give physicians advice about building trust with parental caregivers during a child's hospital admission, what would you say is the most important thing they could do to build trust?

11. What else about your experiences with building trust with physicians have we not talked about yet today?

12. In closing, can you please define trust in the context of physician-parental caregiver relationships?
