# Supplementary material for: Trust of inpatient physicians among parents of children with medical complexity: a qualitative study
Source: Front Pediatr. 2024 Sep 27;12:1443869. doi: 10.3389/fped.2024.1443869 (PMC11466756; doi:10.3389/fped.2024.1443869)
Supplement: Supplementary file 2 [file Datasheet2.pdf]

Standard Operational Definition for Children with Medical Complexity

Provincial Council for Maternal and Child Health
building a brighter future

To be eligible for Complex Care for Kids Ontario, the child must:

- ☐ Be under 18 years of age.
- ☐ Meets one criterion (indicated by a check box) from <u>four of the five</u> categories below.
- ☐ Medically complex child/youth not currently being followed by a multi-disciplinary team (e.g., diabetes team, cystic fibrosis, or neuromuscular clinics). Rather, child/youth should continue to be followed in their current team rather than (individual exceptions aside) referred to the Complex Care for Kids Ontario.

Technology Dependent and/or Users of High Intensity Care

- ☐ Child is dependent on mechanical ventilators, and/or requires prolonged IV administration of nutritional substances or drugs and/or is expected to have prolonged dependence on other device-based support

  For example:
  tracheostomy tube care, artificial airway, suctioning, oxygen support, or tube feeding

- ☐ Child has prolonged dependence on medical devices to compensate for vital bodily functions, and requires daily/near daily nursing care

  For example:
  cardiorespiratory monitors;
  renal dialysis due to kidney failure

- ☐ Child has any chronic condition that requires great level of care such as:
  - Child is completely physically dependent on others for activities of daily living (at an age when they would not otherwise be so dependent)
  - Child requires constant medical or nursing supervision or monitoring, medication administration and/or the quantity of medication and therapy they receive

Fragility

- ☐ The child has severe and/or life-threatening condition
- ☐ Lack of availability and/or failure of equipment, technology, or treatment places the child at immediate risk resulting in a negative health outcome
- ☐ Short-term changes in the child's health status puts them at immediate serious health risk

  For example:
  an intercurrent illness

- ☐ As a consequence of the child's illness, the child remains at significant risk of unpredictable life-threatening deterioration, necessitating round-the-clock monitoring by a knowledgeable caregiver
- ☐ Likely to experience exacerbation of chronic condition necessitating assessment by a healthcare provider in a timely manner

Chronicity

- ☐ The child's condition is expected to last at least six more months
- ☐ The child's life expectancy is less than six months

Complexity

- ☐ Involvement of at least five healthcare practitioners/ teams and healthcare services are delivered in at least three of the following locations:
  - Home, School/ Nursing school
  - Hospital,
  - Children's Treatment Centre,
  - Community-based clinic (e.g. doctor's office)
  - Other (at clinician's discretion)
- ☐ The family circumstances impede their ability to provide day-to-day care or decision making for a child with medical complexity

  For example, the primary caregiver and/or the primary income source are at risk of not being able to complete their day-to-day responsibilities

Geography

- ☐ Child meets criteria for at least three of the four previous categories, and has significant challenges to seek appropriate medical services based on rurality or access

January 2019
