# Supplementary material for: Trust of inpatient physicians among parents of children with medical complexity: a qualitative study
Source: Front Pediatr. 2024 Sep 27;12:1443869. doi: 10.3389/fped.2024.1443869 (PMC11466756; doi:10.3389/fped.2024.1443869)
Supplement: Supplementary file 3 [file Datasheet3.pdf]

Supplementary Material 3

Forming and Maintaining Trust between Physicians, Children with Medical Complexity, and their Parents: A Qualitative Study of Parent Perspectives

Tammie Dewan,[1] Andrea Whiteley,[1] Lyndsay Jerusha MacKay,[2] Rachel Martens,[3] Melanie Noel,[4] Chantelle Barnard[1], Isabel Jordan,[5] Anne Janvier,[6] Sally Thorne[7]

*** Corresponding Author: Tammie Dewan**

Correspondence: tammie.dewan@ucalgary.ca

Nvivo Coding Tree

UNIVERSITY OF CALGARY

Definition of Trust

- Feeling or intuition
- Difficult to define
- Takes time to establish
- Showing warmth and concern
- Decreases stress
- Safe person or place
- Belief that someone is telling the truth
- Helps to take risks or move forward
- Reciprocated
- Knowing someone is doing their best
- Essential if you are vulnerable
- Collaborative relationship
- Someone you can rely on
- When expectations are met

Definition of Trust cont.

- When patient/parent has agency, dignity
- Has your/child's best interest in mind

How parents gain or maintain physician's trust

- Advocate for child
- Allow student training
- Ask questions
- Be honest and open with physicians
- Be confident with staff and physicians
- Be prepared for appointments
- Be present at hospital
- Show competence in caring for child
- Child's records organized & available
- Listen to physician
- Medical understanding
- Parent's make trust a priority
- Monitor child closely and honestly
- Show gratitude and appreciation
  - For child's recovery
  - For healthcare staff
  - For physician
- Welcome feedback and direction on care

How parents gain or maintain physician's trust

- Deal with concerns promptly
- Embrace or accept situation
- Show compassion for care team
- Control (negative) emotions
- Respect medical staff and space

How parents decrease physicians' trust

- Ask for different physician
- Confrontational behaviour
- Do not participate in care of child
- Exaggerate signs or symptoms
- Inability to monitor or assess child
- Overuse of resources
- Overuse of medical terms/ measures
- Question physician's competence
- Unnecessary ED visits

How parents actively increase their trust in physicians

- Focus on medical situation
- Ignore personality differences
- Acknowledge difficulty of situation
- References from others

Advice for new physicians to gain trust

- Acknowledge parents
- Admit you don't know
- Don't take detailed history in emerg
- Be personable
- Introductions important
- Know child's chart
- Take time with first meeting

- Complex Care Relationship
- Expectations
- Personality
- Parent Knowledge
- General Expressions of Gratitude

Nvivo Coding Tree cont.

UNIVERSITY OF CALGARY

How physicians gain parents' trust

- Advocacy
- Approach to care
- Child-physician interaction
- Communication
- Compassion or concern
- Consistency
- Fallibility
- Honesty and truthfulness
- Most important thing
- Partnership
- Physician knowledge
- Relationship
- Time
- Trust building a priority

→ See next slide

Traumatic experiences or incidents

- Child in pain
- Emergency situations
- High level stress reactions
- Medical errors
- Misdiagnosis
- Near-death experience
- Premature or difficult birth
- Stress of long admission for child

Nursing and other health care worker interaction

- Loss of trust in nursing
- Medication issues

Parent Vulnerability

- Scared to ask questions
- Burnout
- Difficult decisions about treatment/care
- Not comfortable to ask for second opinion
- Overwhelmed - child's symptoms/ suffering
- Parents' lives turned upside down
- Self-blame in parents
- When trust is broken
- Dreading future treatment

Loss of Trust in Institution or Health Care

- Lost trust in physician = lost trust in system
- Medical Mistakes
- Miscommunication between depts & staff
- Misdiagnosis or taking long time to diagnose
- Staff shortages
- Staffing issues
- Wait times
- Lack of supplies for child's care
- Payment required for information
- Parent has to provide care for child

How physicians decrease parents' trust

- Unable to predict outcomes
- Unaware of parent's stress levels
- In a rush
- Provide inaccurate info
- Treat other HCP poorly
- Poor listening
- Poor communication skills
- Lack experience or knowledge
- Lack of empathy
- Lack of collaboration with care team
- Offload responsibility on others

How physicians decrease parents' trust continued

- Overly authoritative
- Comment on child's length of life
- Confrontational
- Create power differential
- Dismissive of parents' concerns
- Disrespect of parents or others
- Emphasize they are expert
- Euphemistic /sugar coating
- Judgmental of parents
- Lack of collaboration with parents
- Personality Issues
  - Physician stereotypes
  - God complex physicians
  - Parents accommodating
  - Personality clashes

How physicians decrease parents' trust continued

- Focus only on test results
- Ignore child or don't engage
- Lack knowledge of chart
- Negative body language
- Not acknowledging parents' struggle
- Not admitting they don't know
- Not looking at big picture
- Not providing test results promptly
- Not sharing child's records
- Not taking time for good care

Nvivo Coding Tree – cont.

How physicians gain parents' trust

UNIVERSITY OF CALGARY

Communication
- Use humour appropriately
- Aware of body language
- Clarity of communication
- Use different formats
- Give parents opportunity to speak
- Level of detail
- Good listening skills
- Open to questions
- Open, candid, straightforward
- Parent needs to communicate to others
- Follow up with info or feedback
- Plain language
- Provide full info before decision made
- Share thoughts and ideas
- Ask parents how want to receive information

Approach to care
- Describe procedures
- Ask permission to carry out procedure
- Consistent interaction with family
- Inclusive decision-making practices
- Knowledge about effects of treatment
- Involve child in treatment
- Tailor care approach to child
- Think outside the box
- Treat child as unique
- Willing to take risks
- Err on side of caution
- Allow parents to care for child at home
- Allow parents to make decisions for child
- Asking for parent's opinion
- Asking how they can improve care/trtmnt

Physician knowledge
- Admit you don't know
- Consult with others
- Knowledge about treatment
- K. of child's chart & history
- K of medical issues
- Parent trusts physician's expertise
- Physician does research for child
- Physician learns from parent
- Physician shares research with parent

Approach to care cont.
- Assess & understand parents' perspective
- Dealing w/issues while inpatient
- Expedites admission or treatment
- Honour parent's requests
- Put child's needs first
- Physician trusts parent

Fallibility
- Parent understands pressure on staff
- Understand personality differences
- Acknowledge difficult diagnosis/treatment
- Acknowledge difficult situations
- Apologize if mistake made
- When physician admits not knowing

Consistency or continuity of care
- Follow through on treatment
- Get back to parents with answers
- Of approach with parents
- Of care team
- Of Physician

Time
- Allow time for diagnosis & treatment
- Take time to assess
- Take time with family
- Take time to communicate
- Trust takes time to build

Relationship
- Family centered care
- With child
- With other health care professional
- With parents

Partnership
- Collaborative approach
- Empowering parents
- Parents part of care team
- Parents included in decision-making
- Take parents' concerns seriously

Honesty and truthfulness
- Tactfulness
- Don't lie to hide an error
- Admitting not knowing
- Don't sugarcoat situation
- Authenticity

Compassion or concern for parents
- Ask about mental health of parents
- Care beyond just words
- Compassion and concern for parents
- Parent does not need counselling
- Understand when parents are stressed

Child-physician interaction
- Engage with child
- Best possible QofL as goal
- Play with child
- Show care and compassion for child
- Understanding and relating to older child

Advocacy
- Having physician take your side
- Physician advocates for treatment
- When another HCP advocates for child

Most important thing
- Trust building a priority

Nvivo Coding Tree - Legend

Top level code

Top level code – no subcodes

Subcode 1

Subcode 2
