# Supplementary material for: Trust of inpatient physicians among parents of children with medical complexity: a qualitative study
Source: Front Pediatr. 2024 Sep 27;12:1443869. doi: 10.3389/fped.2024.1443869 (PMC11466756; doi:10.3389/fped.2024.1443869)
Supplement: Supplementary file 4 [file Datasheet4.pdf]

## Supplementary Materials 4

## Research Protocol

## Post-traumatic stress in parents of children with medical complexity and association with parent experiences in hospital

**Excerpt from V5 - 3 January 2023:**

**Study Population**

Potential participants will include parents and primary caregivers of a child between the ages of 6 months to 17 years with medical complexity hospitalized at Alberta Children's Hospital (ACH)..

The definition for a CMC was adapted from the Complex Care Kids Ontario (CCKO) standard operational definition [CCKO webpage] as follows:

1. Child is dependent on medical technology at home (prior to the index hospitalization). Eligible technologies include: feeding tube, oxygen, suction, cardiorespiratory monitor, non-invasive ventilation, tracheostomy, vascular access device, ventriculoperitoneal shunt, vagal nerve stimulator, dialysis.

2. Child's care requires involvement of at least five healthcare practitioners/teams and health care services in the health care or community setting (prior to index hospitalization)

3. Child has significant fragility as evidenced by at least two prior hospital admissions and/or at least one admission to the Pediatric Intensive Care Unit (PICU) or Neonatal Intensive Care Unit (NICU) prior to the index hospitalization.

These inclusion criteria will be screened based on chart review and supplemented with directed questions to a member of the child's care team.

Potential participants must be able to communicate in English. Up to two parents or primary caregivers can participate for each child.

Eligible hospitalizations will have an anticipated duration of greater than three days to allow time for enrollment and baseline evaluation. This assessment will be based on the "Expected Duration of Admission" that is indicated in the admission physician orders and/or based on directed questions to the care team during screening for eligibility. Hospital admissions for suspected non-accidental injury or neglect are ineligible.

**Recruitment**

Although hospital admissions are a time of stress for parents, these are also key opportunities to identify and recruit parents of CMC. During hospitalizations, parents may have assistance caring for their children and thus more flexibility to participate in research activities. Potential participants will be

identified through speaking with the charge or bedside nurse on each unit and/or daily review of handover lists of all teams at Alberta Children's Hospital for units 2, 3, 4 and the PICU. After a charge or bedside nurse confirms potential eligibility, formal screening for eligibility will be conducted by review of the hospital chart supplemented by discussion with members of the care team where necessary to confirm eligibility [APPENDIX – Screening Checklist]. Potential participants will be identified as early in the hospitalization as possible to allow time for recruitment and data collection procedures. Eligible parents or primary caregivers will be approached by a familiar member of their clinical team (physician, nurse or allied health) to introduce the study and ask for permission for the study team to make contact. This contact will take place by phone or in person if allowed in the current COVID restrictions. The research assistant will provide verbal and written information about the study, as well a copy of the consent form if they are interested in proceeding. The signed consent form may be received either in hardcopy or via REDCap. The participant will be given up to 3 days to review the consent form and decide if they want to enroll in the study. This timeline is truncated due to the goal for enrollment and data collection to occur during the admission. When parental caregivers are admitted to the study during hospitalization and have signed study consent forms, but are unable to fill out the baseline questionnaires before discharge or are not admitted for a minimum of 3 days, parental caregivers will remain enrolled in the study and be approached to fill out the baseline questionnaires during their child's next admission.
